# Next-Generation Pharmacotherapy for Uterine Leiomyomas: Combining Metformin and Ulipristal Acetate as Neoadjuvant and Adjuvant Interventions to Minimally Invasive Procedures

**DOI:** 10.3390/life16101676

**Published:** 2026-10-07

**Authors:** Nuray Rozmurat, Gulzhanat Aimagambetova, Sanja Terzic, Peng Zhao, Gauri Bapayeva, Kuralay Kongrtay, Matthew Naanlep Tanko, Ainur Abdrakhmanova, Antonio Simone Laganà, Milan Terzic

**Affiliations:** 1 School of Medicine, Nazarbayev University, Zhanybek-Kerey Khans Street 5/1, Astana 010000, Kazakhstan; nuray.rozmurat@nu.edu.kz (N.R.); aabdrakhmanova@alumni.nu.edu.kz (A.A.); 2 Department of Surgery, School of Medicine, Nazarbayev University, Zhanybek-Kerey Khans Street 5/1, Astana 010000, Kazakhstanmilan.terzic@nu.edu.kz (M.T.); 3 Clinical Academic Department of Women’s Health, CF “University Medical Center”, Turan Ave. 32, Astana 010000, Kazakhstan; gauri.bapayeva@gmail.com; 4 Department of Medicine, School of Medicine, Nazarbayev University, Zhanybek-Kerey Khans Street 5/1, Astana 010000, Kazakhstan; sanja.terzic@nu.edu.kz (S.T.); matthew.tanko@nu.edu.kz (M.N.T.); 5 Assisted Reproduction Unit, Department of Obstetrics and Gynecology, Sir Run Run Shaw Hospital, School of Medicine, Zhejiang University, Hangzhou 310016, China; zhaopeng0622@zju.edu.cn; 6 Zhejiang Provincial Clinical Research Center for Reproductive Health and Disease, Hangzhou 310016, China; 7 Zhejiang Key Laboratory of Precise Protection and Promotion of Fertility, Hangzhou 310016, China; 8 Obstetrics and Gynecology Unit, Ospedali Riuniti Villa Sofia-Cervello, Department of Health Promotion, Mother and Child Care, Internal Medicine and Medical Specialties (PROMISE), University of Palermo, 90121 Palermo, Italy; antoniosimone.lagana@unipa.it

**Keywords:** uterine leiomyoma, uterine fibroids, metformin, ulipristal acetate

## Abstract

Uterine fibroids (leiomyomas) are the most common benign tumors of the myometrium in women of reproductive age. They are a major cause of heavy menstrual bleeding, pelvic pressure and pain, and reduced fertility in women of reproductive age. This scoping review aims to examine the potential of two existing medications, metformin and ulipristal acetate (UPA), to treat fibroids individually and in combination with current minimally invasive procedures. Studies published between January 2016 and May 2026 were identified from four databases: PubMed, Scopus, EBSCO, and Google Scholar. Of the 2583 papers identified, 23 studies met the inclusion criteria for this review. Ulipristal acetate (UPA) has been shown to decrease heavy menstrual bleeding and uterine fibroid volume in several clinical studies. However, its use as a treatment for uterine fibroids has been severely restricted in several countries because of the rare occurrence of idiosyncratic drug-induced liver injury. Metformin acts on multiple pathways reported to be involved in the pathogenesis of leiomyomas, including activation of the energy sensor AMP-activated protein kinase (AMPK), inhibition of the mammalian target of rapamycin (mTOR) signaling pathway, modulation of steroidogenesis, suppression of angiogenesis, and induction of apoptosis. The complementary molecular mechanisms of action of metformin and UPA provide a theoretical basis for investigating combination therapy. However, there is currently a lack of direct evidence regarding the potential for synergistic reduction in fibroid volume or regression with combination treatment, and no clinical trials have investigated the use of metformin in conjunction with UPA for the treatment of uterine fibroids in humans. Future studies of metformin and UPA may also benefit from the development of targeted intrauterine and intralesional delivery systems to increase local drug exposure. However, the pharmacokinetics, tissue penetration, hepatic safety, and clinical efficacy of such systems remain to be established. Therefore, while the available evidence supports further investigation of metformin and UPA individually as potential therapeutic agents for uterine fibroids, their combined use, as well as their use in conjunction with current minimally invasive procedures for fibroid treatment, should be regarded as investigational and requires further preclinical and prospective clinical evaluation.

## 1. Introduction

Uterine leiomyomas (fibroids) are the most common benign smooth muscle tumors of the uterus and arise from monoclonal transformation of myometrial smooth muscle cells. Many affected women experience menorrhagia, pelvic pain, dysmenorrhea, and low back pain, resulting in substantial physical, reproductive, and psychological burden [1,2,3,4,5]. Clinical manifestations vary according to fibroid type (submucosal, intramural, or subserosal), size, and associated complications such as bleeding, degeneration, infection, and inflammation [6]. In pregnancy, fibroids are associated with increased risks of preterm labor, placental abruption, fetal malpresentation, premature rupture of membranes, cesarean delivery, postpartum hemorrhage, and fetal growth restriction [7]. Intramural and submucosal fibroids may also impair fertility and assisted reproductive outcomes, with submucosal fibroids adversely affecting reproduction regardless of size [8,9,10].

Risk factors for uterine leiomyoma include age, race, early menarche, hormonal exposure, obesity, uterine infection, lifestyle factors, family history, vitamin D deficiency, hypertension, and exposure to environmental endocrine-disrupting chemicals [3,11].

**Figure 1:**
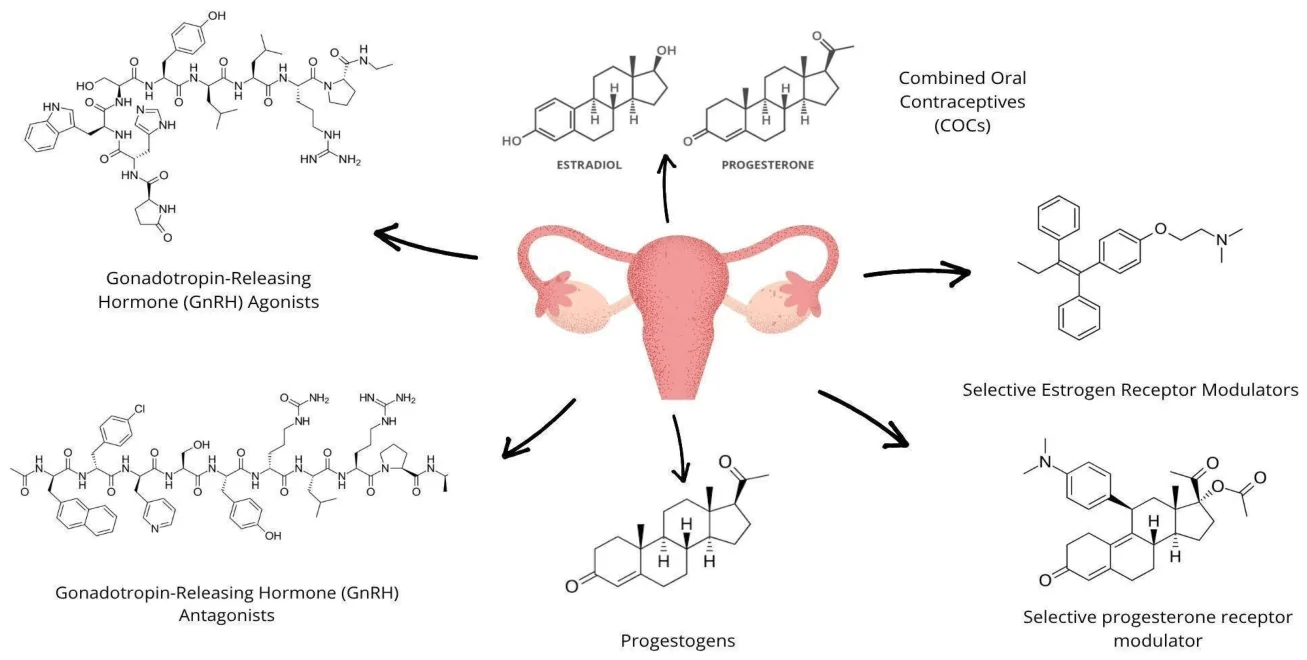
Pharmacological management of uterine leiomyomas (created using Canva). The figure provides an overview of currently used medical therapies for uterine leiomyomas including combined oral contraceptives, progestogens, selective estrogen receptor modulators (SERMs), selective progesterone receptor modulators (SPRMs) and GnRH agonists/antagonists with their respective main effects.

Current treatment options include radiological, surgical, and pharmacological approaches, with management individualized according to symptoms, fibroid characteristics, and fertility goals [12,13]. Medical therapy is particularly useful for women with heavy menstrual bleeding or pain who wish to preserve fertility and may also be used preoperatively to correct anemia and reduce fibroid size [12,14]. Pharmacological options include combined oral contraceptives, selective estrogen receptor modulators, progestogens, selective progesterone receptor modulators, and GnRH agonists and antagonists. These treatments primarily aim to control heavy menstrual bleeding and temporarily reduce fibroid volume, although their long-term use may be limited by adverse effects [15]. Current medical treatment options are summarized in Figure 1.

There are six primary medical therapies that influence uterine fibroid development and management: COCs, SERMs, progestogens, SPRMs, GnRH agonists, and GnRH antagonists. COCs and progestogens are used for short term, but they are good to manage heavy menstrual bleeding [1,12,16,17]. SERMs decrease heavy bleeding, trigger amenorrhea, and improve the quality of life [12,13,18,19,20]. In general, COCs and SERMs have limited evidence on reducing fibroid size [16,21]. GnRH agonists and antagonists are effective in reducing fibroid volume and heavy menstrual bleeding [22,23,24,25]; however, they are used only short-term and mainly before surgery, to avoid adverse effects such as hot flushes, reduced vaginal lubrication, and reduced bone mineral density Figure 1 [12,17].

Metformin is currently the most prescribed anti-diabetic drug, to treat type 2 diabetes. Except for its use in diabetes treatment, other useful features such as antioxidant, anti-inflammatory, and anti-tumor effects were identified. Recent research has explored its influence on leiomyoma growth [24].

Due to its low cost and well-established safety profile, metformin is considered a strong therapeutic option focused on prevention and clinical management of gynecological disorders, including endometriosis and uterine fibroids. Metformin (N,N-dimethyl biguanide produces specific growth-inhibitory effects myometrial regulatory pathways serving as an essential metabolic regulator [25]. SPRM, ulipristal acetate, reduces leiomyoma in fibroid tissue by controlling ECM, supporting apoptosis and reducing cell growth [26].

Our scoping review focused on the emerging role of metformin and UPA in the management of uterine leiomyomas. The primary objective of this scoping review is to assess the evidence available on metformin and ulipristal acetate including therapeutic potential, mechanism of action, efficacy, safety and relevance to medical treatment of uterine leiomyomas. This review does not analyze the compounds separately or focus only on their individual modes of action as promising therapeutic agents. Instead, it compares their intracellular target pathways to identify shared molecular features. In this review, the mechanisms of action of these compounds are integrated into a unified therapeutic framework for benign gynecological disease and other locally advanced diseases, with emphasis on their potential applications in nonsurgical treatment strategies.

## 2. Materials and Methods

### 2.1. Arksey and O’Malley Framework

The review was conducted within the framework provided by Arksey and O’Malley [27] and was conducted in strict adherence to the Preferred Reporting Items for Systematic Reviews and Meta-Analyses extension for Scoping Reviews (PRISMA-ScR) guidelines. The review systematically searched, mapped, characterized and interpreted literature that examines emerging medical therapies for uterine leiomyomas, with reference to metformin and ulipristal acetate. The Arksey and O’Malley methodological framework outlines a five-stage process to be followed when conducting a scoping review: identifying the research question, identifying the relevant studies, selecting the eligible studies, charting the data and collating, summarizing and reporting the results. As it is a scoping review, no formal critical appraisal of the included studies was performed, and no risk-of-bias conclusions are provided. The diversity of study designs, patient populations, and clinical studies did not allow for a reliable assessment. Therefore, the scoping review concentrates on mapping the existing and available literature, indicating the need to observe human clinical studies with co-treatment of UPA and metformin.

### 2.2. Identifying Research Questions

The primary research question was: What evidence exists on the effectiveness, mechanisms, and safety of metformin and ulipristal acetate in the medical management of benign uterine leiomyomas?

### 2.3. Eligibility Criteria

The criteria for inclusion and exclusion for this scoping review are specified and align with the PRISMA framework. This review includes only published, peer-reviewed studies retrieved from the online databases of PubMed, Scopus, EBSCO and Google Scholar. Selection criteria for inclusion in the review were (a) studies related to benign uterine leiomyomas or uterine fibroids; (b) studies that investigated the use of metformin or ulipristal acetate for medical management of uterine leiomyomas; (c) preclinical or clinical research on growth, bleeding, symptom relief, and/or mechanisms of action, recurrence, side effects, and quality of life and fertility-related outcomes for medically managed uterine fibroids; (d) primary research that included both qualitative and/or quantitative methodologies (including experimental and observational studies or a combination of both study designs); and (e) studies published in the English language. This scoping review did not include studies on other types of leiomyomas, except uterine leiomyomas. Studies published between January 2016 and May 2026 were included to identify the most recent decade of evidence and reflect contemporary developments, practices, and research within the field. The review also excluded studies on the use of metformin and ulipristal acetate for conditions other than fibroid management in reproductive health or gynecology. This scoping review does not include literature reviews, grey literature, and non–peer reviewed material. Only articles published between January 2016 and May 2026 were included in the review. Details of the search strings, search parameters, and inclusion criteria are listed in Table 1.

### 2.4. Search Strategy and Selection Process

The search terms for this scoping review were developed from the words and abbreviations related to the research question. Specifically, “Leiomyoma” (MeSH Unique ID: D007889), or “uterine leiomyoma”, “uterine fibroid”, “fibroid”, and the medications “Metformin” (MeSH Unique ID: D008687), and “ulipristal acetate” (MeSH Supplementary Concept Unique ID: C555622), “UPA”, and “selective progesterone receptor modulator” were used to search the literature. In addition, words and aliases such as “leiomyoma,” “myoma,” “fibroid tumors,” “metformin,” “uterine leiomyomas,” and “uterine fibroids” were included to capture relevant literature that uses different terms to describe the condition and the medications of interest. Two reviewers independently went through the title and the abstract of all records identified through the literature search. Subsequently the full text of the records that seemed to be included were checked for final inclusion. Any disagreements that occurred during the screening process were resolved through discussion between the two reviewers. If necessary, a third reviewer was brought in for discussion.

For PubMed, the search used MeSH terms for uterine leiomyomas, metformin and ulipristal acetate: (“Metformin” [Mesh] OR metformin) AND (“Leiomyoma” [Mesh] OR “uterine leiomyoma” OR “uterine fibroid” OR fibroid) (“Ulipristal Acetate” [Mesh] OR “ulipristal acetate” OR ulipristal OR UPA) AND (“Leiomyoma” [Mesh] OR “uterine leiomyoma” OR “uterine fibroid” OR fibroid).

For EBSCO, Google Scholar and Scopus used keyword-based search strings, including terms related to metformin, ulipristal acetate, and uterine fibroids (leiomyomas).

## 3. Results

### 3.1. Overview of Included Studies

**Figure 2:**
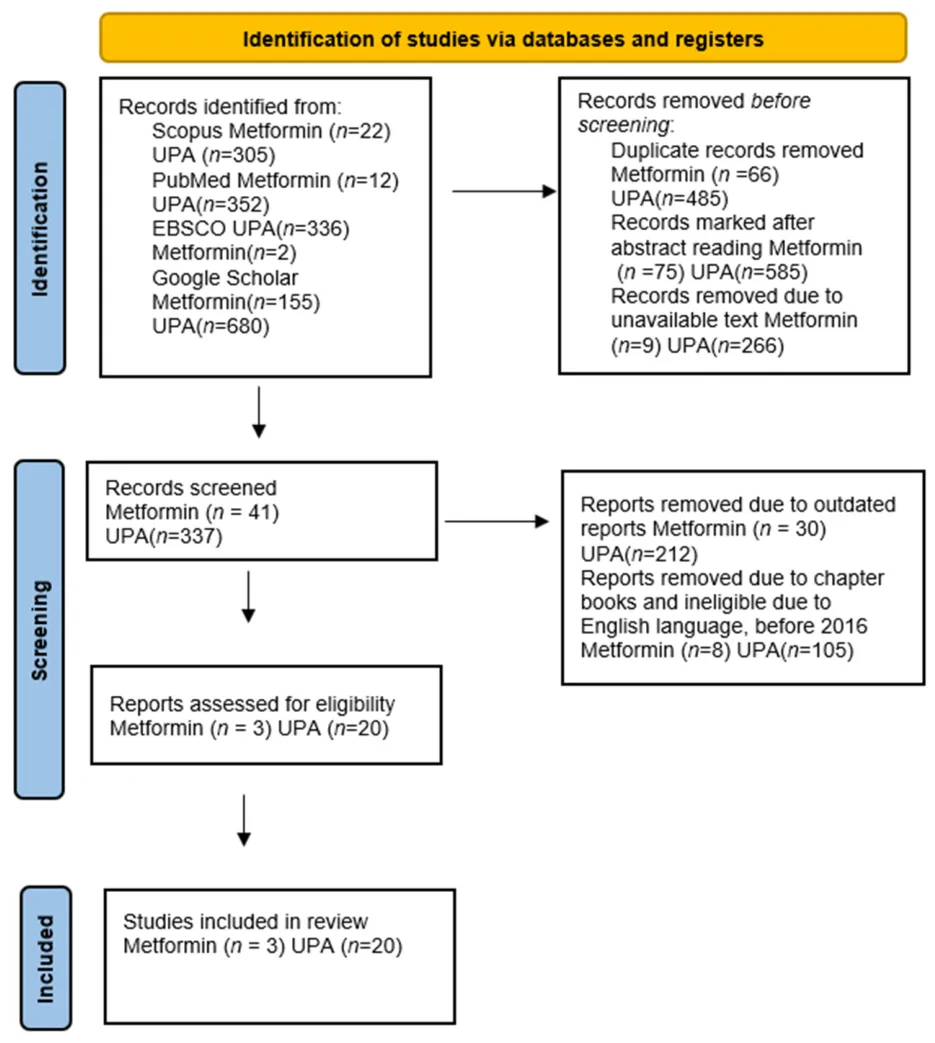
Data collection flowchart (using Prisma guideline).

After screening the identified records, a total of 23 studies (20 studies for UPA, 3 studies for Metformin) met the inclusion criteria and were included in this scoping review (Figure 2). Most of the studies reviewed were from Europe and Asia. In addition to studies from Europe, there were also included studies from Asia such as Japan, Taiwan, Korea, and India. The types of studies included in the review were case–control studies, retrospective cohort studies, prospective cohort studies, randomized controlled trials as well as prospective clinical studies. The number of study patients ranged from 10 to 21,996 patients. All study participants were adult women and their age distributions varied across the included studies. While several studies primarily included women in their reproductive years, other investigations enrolled broader age groups or midlife women. Therefore, participant age is reported separately for each study in the corresponding study characteristics table. The included studies were categorized into two main groups. The first group consisted of observational studies that investigated the association between diabetes or metformin exposure and the risk of developing leiomyoma. The second group included the interventional studies that investigated the use of ulipristal acetate for the treatment of women with symptomatic uterine fibroids.

### 3.2. Data Extraction and Charting for Metformin Studies

The search strategy applied resulted in the identification of three studies that addressed diabetes or metformin exposure. Studies on diabetes or metformin exposure suggested that patients with type 2 diabetes or taking metformin had lower odds of developing uterine leiomyoma; some studies suggested a reduced risk [24,28,29].

These studies were observational and did not directly evaluate metformin as a therapeutic intervention for uterine leiomyomas. Their findings should therefore be interpreted as associations with fibroid risk or diagnosis rather than evidence of treatment efficacy (Table 2).

### 3.3. Data Extraction and Charting for Ulipristal Acetate (UPA) Studies

UPA has been evaluated in 20 clinical studies, including single and repeat dosing studies [30,31,32,33,34,35,36,37,38,39,40,41,42,43,44,45,46,47,48,49]. In these randomized and prospective clinical studies involving women with uterine fibroids, ulipristal acetate (provided high effectiveness in stabilizing circulatory stability, producing successful results of early and sustained amenorrhea). The treatment led to significant clinical improvements in health-related quality-of-life scores and promoted substantial regression in tumor volume. Effective relief of symptoms of uterine fibroids was observed in multiple cycles of treatment with this drug [20,21,22,23,24,25,26,27,28,29,30,31,32,33,34,35,36,37,38,39,40,41,42,43,44,45,46,47,48,49].

Side effects of ulipristal acetate were observed in the clinical studies and included hot flushes, headache, nausea, constipation, menorrhagia, metrorrhagia, genital bleeding, abdominal pain, and back pain [30,31,32,33,37,38,43,44,45,46,47,48,49]. Adverse effects associated with metformin could not be evaluated from the included observational studies because these studies did not assess or report treatment-related adverse events. Very large heterogeneity existed between studies [24,28,29]. Differences existed between studies in study design, patient population, dose of fibroid intervention, duration of treatment, and outcomes (Table 3).

### 3.4. Key Findings

**Figure 3:**
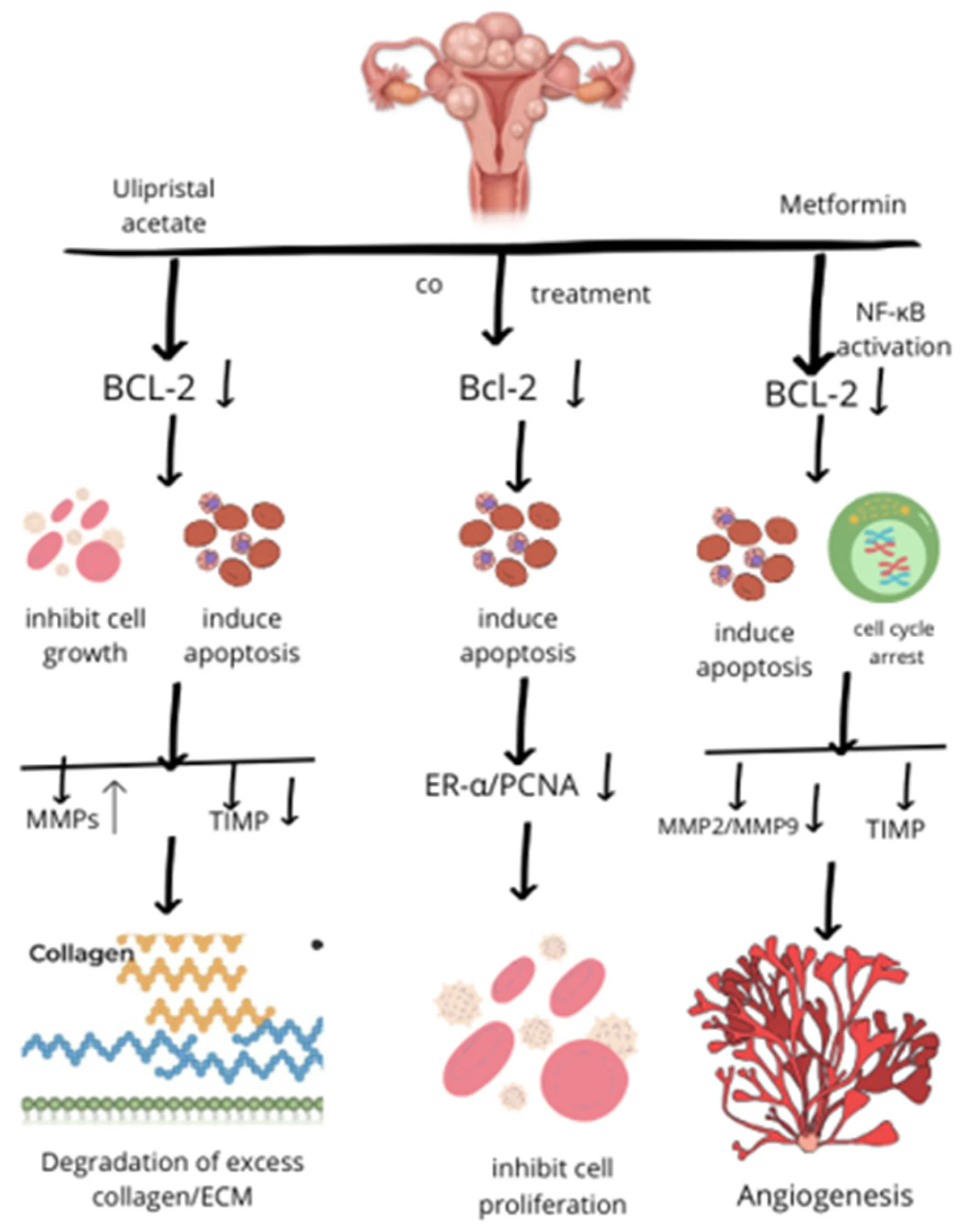
Synergistic pathways of metformin and UPA in uterine fibroids (created using Canva). The figure illustrates synergistic pathways including modulation of the AMPK/mTOR pathway, blockade of progesterone receptor action, inhibition of cell proliferation and induction of apoptosis, inhibition of extracellular matrix deposition, and effects on uterine fibroid angiogenesis.

Uterine fibroids originate from multiple treatment mechanisms, which include metabolic-growth pathways, ovarian steroid signaling, extracellular matrix accumulation and angiogenesis. Some treatments can be mediated either alone or with a combination treatment [1,2,3,4,5]. A combined approach using metformin and UPA may be hypothesized because these two agents act through different primary pathways but may interact on common anti-leiomyoma endpoints. For example, the potential of the combination of metformin and UPA to enhance anti-fibroid effects was investigated in a rat model. The treatment caused a shift in the Bax/Bcl-2 ratio toward apoptosis and decreased the expression of the proliferation marker genes PCNA and Cyclin D1 leading to inhibition of cell proliferation. In addition, the treatment decreased estrogen signaling through ER-α (Figure 3) [32].

Figure 3 illustrates the possible synergistic actions of metformin and UPA in relation to the respective overlapping pathways. Thus, a novel therapeutic strategy could be created for the treatment of uterine fibroids by using metformin and UPA in combination.

### 3.5. Effect of Metformin on Uterine Leiomyoma (Fibroids)

The primary impact of the metformin on uterine leiomyoma is inhibition of cell growth through the activation of the energy sensor AMPK, which restrains the mTOR pathway [50]. AMPK activation reduces cell growth by activating TSC2, which works with TSC1 to block the mTOR pathway. Metformin may promote apoptosis and suppress cell proliferation by inhibiting NF-κB signaling and modulating Bcl-2 expression [24,51]. (Figure 3). In addition to TSC2-dependent effects, metformin may suppress leiomyoma growth through TSC2-independent mechanisms. The ELT-3 uterine leiomyoma cell line is deficient in TSC2 and does not express TSC2 mRNA or protein. However, metformin still inhibited the growth of ELT-3 cells. This indicates that metformin may also suppress leiomyoma cell growth through a TSC2-independent pathway [25].

Metformin may restrict fibroid growth not only through the AMPK/mTOR pathway but also by affecting pathways related to estrogen [52]. Primary experimental evidence has shown that metformin reduces aromatase expression in human uterine leiomyoma cells, suggesting that suppression of local estrogen production may contribute to its antiproliferative effects. The enzymes 17β-HSD and aromatase change androstenedione into estrone. Aromatases (estrogen synthases) are enzymes found in greater concentrations in fibroid tissue than in normal myometrium. Metformin causes the decrease in aromatase expression in fibroid cells which leads to the decrease in local estrone production and to the slowing down of fibroid growth [25]. Metformin affects the cells’ metabolic pathways controlling the use of amino acids and causes the cells to cycle and arrest in the G0/G1 or S phase of the cell cycle. This prevents cells from continuing to divide and may induce apoptosis. Metformin may also reduce angiogenesis by lowering MMP2 and MMP9 expression and increasing TIMP levels [53] (Figure 3).

### 3.6. Effect of Ulipristal Acetate (UPA) on Uterine Leiomyoma (Fibroids)

UPA has been reported to reduce proliferation in primary cultured leiomyoma cells [54]. It may slow leiomyoma growth by affecting cell cycle regulators such as p21 and p27, as well as ECM-related molecules including MMP-2 and MMP-9. UPA also lowers BCL-2 gene expression, which may reduce cell growth and promote apoptosis. UPA may also affect MMPs and TIMPs and reduce type I and type III collagen synthesis. Since these factors are involved in abnormal ECM production, future treatments may focus on controlling ECM formation and breakdown, such as by increasing TIMPs and reducing MMPs [55].

Versican (VCAN) is a large chondroitin sulfate proteoglycan that helps form physical barriers and fill spaces in tissues. It also plays an important role in cell migration, cell proliferation, cell positioning, and nerve growth [56]. In fibroid tissues treated with placebo, ECM proteins such as collagen, fibronectin 1 (FN1), and versican were found at higher levels than in normal uterine muscle from the corresponding patients [57]. Clinical studies have shown that collagen type I is the most common fibrillary collagen and makes up a large part of the disordered ECM in uterine fibroids. After long-term treatment with UPA, collagen levels decreased. However, short-term treatment for 3 months did not change collagen levels [58]. Also, it decreases neo-angiogenesis by inhibiting expression of VEGF [59].

Microarray analysis showed that several versican isoforms were increased in leiomyoma tissue and leiomyoma cells. Clinical studies found that versican levels decreased more after UPA treatment than in the placebo-treated group. UPA treatment reduced versican protein levels in leiomyomas, and these fibroids treated also became smaller. The patients with the largest reduction in total fibroid volume, also showed the greatest decrease in versican protein expression [55].

## 4. Discussion

For uterine leiomyomas, two such potential medications to be studied are metformin and ulipristal acetate. Metformin is an oral medication prescribed to type 2 diabetes patients and has effects on hormone levels as well as modulating the AMPK/mTOR pathway and can cause anti-angiogenesis [24,28,29]. Few studies have investigated the use of these two drugs for the treatment of uterine leiomyomas. Most studies are in vitro studies or observational studies and suggest promise for the treatment of uterine leiomyomas. Human evidence for the use of metformin in the treatment of fibroids is currently very limited. All the studies included in this review were observational in nature and looked at the association between diabetes or exposure to metformin and the risk of developing uterine leiomyomas, or the diagnosis of uterine leiomyomas. Thus, there is no human evidence to support the use of metformin to decrease the volume of fibroids or to improve symptoms related to fibroids. However, there is some mechanistic and preclinical evidence to support further investigation of metformin for fibroids. Mostly Ulipristal acetate has been shown to cause a reduction in size of the uterine leiomyomas with potential to decrease symptoms and reduce hyperinsulinemia and decrease angiogenesis by decreasing expression of VEGF [59] (Table 4).

However, there are limited large clinical trials in humans. Although UPA is effective in controlling heavy menstrual bleeding, reducing fibroid size, improving quality of life, and providing symptom relief for patients, it is restricted in several regions due to its risk of liver injury [60]. Preclinical evidence for combined metformin and UPA therapy is limited to a rat study assessing metformin protection against UPA-induced endometrial changes. Therefore, the combination should be regarded as hypothesis-generating, and no human clinical trial has yet evaluated this approach.

### 4.1. Mechanistic Pathways of Ulipristal Acetate and Metformin in the Endometrium

UPA induces micromorphological and functional changes in the endometrium by binding to local progesterone receptors with an affinity like that of progesterone. This blocks progesterone-dependent endometrial proliferation during the menstrual cycle, leading to rapid amenorrhea and reduced menstrual bleeding [44]. Long-term UPA use is associated with several systemic effects. In some cases, systemic exposure may cause benign endometrial changes, such as progesterone receptor modulator-associated endometrial changes (PAEC), which are typically non-atypical and usually reversible [61].

In contrast, the effects of metformin on the endometrium are based on its metabolic and antiproliferative actions. Activation of endometrial AMPK by metformin improves local insulin sensitivity and inhibits mTOR signaling, thereby sustaining endometrial homeostasis. The metformin also upregulates genes important for endometrial homeostasis as well as preparation for implantation such as homeobox gene A10 (HOXA10) and glucose transporter 4 (GLUT4) [62].

The mechanistic evidence supporting metformin should also be interpreted with caution. Much of the evidence is derived from studies of non-leiomyoma tissues or disease models [50,53,59,62], with limited evidence specifically from leiomyoma cells [51].

Overall, the effects of UPA are thought to be largely receptor-mediated, whereas the effects of metformin involve a broader range of targets. These targets are related to systemic metabolic pathways, antiproliferative actions, and endometrial receptivity parameters [24,37].

### 4.2. Future Therapeutic Perspectives: Localized Co-Delivery, Amplified Signal Intersection, and Integration with Minimally Invasive Interventions

Leiomyomas can be managed with a number of effective pharmacological therapies; however, many of these treatments are associated with side effects and high levels of systemic exposure [63]. UPA has been shown to decrease both uterine bleeding associated with leiomyomas and the size of individual fibroids. However, due to reports of rare but serious idiosyncratic drug-induced liver injury (DILI), including cases requiring liver transplantation, the use of UPA has been heavily restricted [60]. In the European Union, UPA is approved for use in premenopausal women with symptomatic leiomyomas for whom surgery or uterine fibroid embolization is unsuitable or has failed. UPA is not indicated for symptom control while awaiting surgery. Metformin may affect pathways that influence leiomyoma growth and development, such as the AMPK/mTOR pathway, which regulates cell proliferation and metabolism [24,25,50,51]. However, the human evidence supporting the use of metformin to reduce leiomyoma size or alleviate symptoms is largely observational and remains insufficient to support its use for these purposes.

The two drugs could potentially be combined to target different molecular pathways. UPA acts through modulation of progesterone receptor activity and effects on extracellular matrix-related pathways [26,54,55]. Metformin activates the AMPK signaling pathway and inhibits mTOR signaling [24,50,51]. However, there is insufficient evidence to support a synergistic effect between the two drugs. The only study investigating a combination of UPA and metformin was performed in rats and examined the effect of metformin on UPA-induced endometrial changes [64]. The study found that the combination altered markers of proliferation and apoptosis, including Bax, Bcl-2, PCNA, and cyclin D1. However, effects on fibroid regression, fibroid-volume reduction, and enhanced cytotoxicity were not investigated; therefore, the combination of UPA and metformin should be considered a hypothesis-generating approach.

In addition to these mechanisms, both UPA and metformin may affect other pathways involved in the growth of uterine leiomyomas. For example, UPA has been reported to modulate extracellular matrix remodeling by affecting matrix metalloproteinase activity [55,58]. Metformin, in turn, may affect various metabolic and proliferative signaling pathways [25,51]. It is therefore of interest to investigate whether simultaneous modulation of these pathways can result in additional therapeutic effects.

Local drug delivery to the uterine cavity using intrauterine devices, hydrogels, or other drug-delivery systems is an investigational approach for the management of uterine leiomyomas. Information regarding the pharmacokinetics, tissue penetration, hepatic safety, and efficacy of locally administered UPA, as well as combined UPA and metformin delivery, is lacking. Furthermore, the risk of idiosyncratic DILI associated with UPA cannot be assumed to be eliminated, and it remains to be determined whether local administration can reduce this risk. It is also important to note that the ability of the intrauterine route to deliver adequate amounts of UPA to intramural and subserosal leiomyomas has not been established. These agents are also being investigated as potential neoadjuvant or adjuvant therapies alongside surgical or ablative procedures, such as uterine artery embolization or HIFU, and such use requires further preclinical and clinical investigation (Table 5).

This new novel approach for an integrative site-specific combined therapeutic approach for fibroids can be translated into clinical management of premenopausal women wishing to preserve their uterus and reproductive capability in the long term. Metabolic syndrome of insulin resistance and PCOS are frequent co-morbid conditions with fibroids and currently available medical therapy for fibroids (GnRH-agonists) are used as hormonal systemic therapy, which in the long-term has undesirable effects of causing or worsening the above-mentioned metabolic disturbances. In the past, powerful fibroid drugs have had safety concerns that precluded their long-term use. Therefore, to maximize benefit of these powerful drugs, it is important to identify the high-benefit patient and in a non-surgical localized and synergistic manner develop new standards of care for management of women with fibroids.

### 4.3. Strengths and Limitations

This scoping review is based on a solid framework and methodology. It is the first multidisciplinary scoping review of cell-based studies investigating the potential of combining SPRMs with biguanides for metabolic restriction. The review may provide new insights into the potentially complementary molecular pathways targeted by these two drug classes. However, whether their combined use produces additive or synergistic therapeutic effects in uterine leiomyomas remains unknown and requires direct preclinical and clinical investigation. By focusing on this dual-therapy approach, the review may help translate basic science into potential clinical applications. Future studies could investigate whether combined targeting of these pathways has potential as a neoadjuvant or adjuvant strategy alongside minimally invasive procedures; however, such applications remain hypothetical and have not been evaluated in clinical trials.

This review includes several limitations. No human clinical trials have investigated the combined effects of metformin and ulipristal acetate in non-diabetic women. Direct evidence on the effects of metformin is very limited and is based mainly on retrospective and case–control studies, in which metformin exposure was assessed as a risk factor. There is several available evidence about ulipristal acetate, but the effect on specific or target tissue is not well discovered. Also, this scoping review does not include risk-of-bias assessment but highlights the developing research in this area.

### 4.4. Recommendation for Future Studies

Further investigation of molecular pathways relevant to uterine leiomyoma treatment should be undertaken in well-designed clinical studies, preferably multicenter randomized controlled trials. The potential combined use of metformin and UPA should be evaluated as a hypothesis-generating strategy rather than an established treatment, with outcomes including fibroid volume, abnormal uterine bleeding, pressure symptoms, and health-related quality of life. Prospective studies of metformin should determine the optimal dose and duration of treatment and identify patient groups most likely to benefit, including premenopausal women with or without metabolic syndrome. Further research is also needed to clarify the roles of insulin resistance and polycystic ovary syndrome in fibroid development.

For UPA, future studies should investigate local delivery approaches, including hydrogel-based and intrauterine systems, with careful assessment of pharmacokinetics, tissue penetration, hepatic safety, and clinical efficacy. Reduced systemic exposure should not be assumed to eliminate the risk of idiosyncratic drug-induced liver injury. Any proposed metformin–UPA combination should also be compared with established treatments, including GnRH agonists and antagonists, using outcomes such as symptom control, patient-reported outcomes, fibroid recurrence, and safety. Although current studies include women from different geographic and ethnic backgrounds, larger and more diverse populations are needed to improve the generalizability of future findings.

More importantly, many in vitro studies have used high concentrations of metformin, ranging from the high micromolar to millimolar range, which are substantially higher than the low micromolar plasma concentrations typically achieved in humans during therapeutic dosing (approximately 10–40 μM). Therefore, the clinical relevance of much of the mechanistic evidence for metformin remains to be established [67].

## 5. Conclusions

This scoping review summarizes the current evidence on metformin and ulipristal acetate (UPA) in the management of uterine leiomyomas. UPA has demonstrated clinical efficacy in reducing fibroid-related bleeding and, in some studies, fibroid volume; however, its use is limited by the risk of rare but serious idiosyncratic drug-induced liver injury and related regulatory restrictions. Metformin may influence several pathways involved in leiomyoma biology, including cellular proliferation, metabolism, angiogenesis, and steroidogenesis, but the available human evidence remains limited and largely observational.

Although metformin and UPA act through distinct molecular pathways, there is currently no direct evidence demonstrating therapeutic synergy or enhanced fibroid regression when the two agents are combined. The available combination evidence is limited to a preclinical rat study that examined UPA-associated endometrial changes rather than leiomyoma regression. Therefore, combined metformin and UPA therapy should be considered a hypothesis-generating strategy that requires further preclinical and clinical evaluation.

Local delivery approaches, including intrauterine and hydrogel-based systems, also remain investigational, as their pharmacokinetics, tissue penetration, hepatic safety, and clinical efficacy have not been established. Likewise, the potential use of these agents as neoadjuvant or adjuvant therapies alongside minimally invasive procedures requires further study before clinical application.

## Figures and Tables

**Table 1:** Research terms and selection criteria.

Term	Associated Term	Exclusions	Inclusions
Uterine leiomyoma	Uterine fibroid, fibroid, myoma, leiomyomata, fibromyoma	Leiomyosarcoma, non-uterine leiomyoma, adenomyosis alone, endometriosis alone	Studies focused on benign uterine leiomyomas/fibroids
Metformin	Dimethylbiguanide, insulin sensitizer, AMPK activator, and antidiabetic agent	Metformin studies unrelated to fibroids or reproductive gynecologic outcomes	Epidemiologic or clinical studies assessing metformin for fibroid growth, symptoms, mechanisms, recurrence, or volume
Ulipristal acetate	UPA, selective progesterone receptor modulator, SPRM.	Emergency contraception studies or ulipristal use unrelated to fibroid treatment	Studies evaluating ulipristal acetate for fibroid bleeding, volume reduction, symptoms, preoperative therapy, safety, or recurrence
Medical therapy outcomes	Pharmacologic treatment, non-surgical management, bleeding control, fibroid volume, quality of life, fertility, safety	Surgical-only or procedural-only studies without medical therapy	Studies reporting clinical, imaging, molecular, safety, or fertility-related outcomes of medical treatment

AMPK activator, AMP-activated protein kinase activator; UPA, Ulipristal Acetate; SPRM, Selective progesterone receptor modulator.

**Table 2:** Observational studies evaluating associations between metformin, diabetes, and uterine leiomyoma risk.

Author/Year	Country	Study Design	Sample Size	Exposure Assessed	Key Findings	Adverse Effects
Velez Edwards et al., 2017 [28]	USA	case–control study	3789 women 608 fibroid cases and 3181 controls	No fibroid-directed intervention	Type 2 diabetes was associated with lower odds of uterine fibroids	Not assessed
Tseng, 2019 [24]	Taiwan	retrospective cohort study	21,996 women with new-onset type 2 diabetes: 10,998 metformin ever-users and 10,998 never-users after matching	No fibroid-directed intervention	Use of Metformin was associated with a lower risk of uterine leiomyoma. Number of cases of leiomyoma in never-users vs. ever-users were 321 vs. 162; incidence rates were 704.65 per 100,000 person-years in never-users and 329.82 in ever-users. The overall HR was 0.467.	Not assessed
Mitro et al., 2025 [29]	USA	Prospective cohort study	2570 midlife women	No fibroid-directed intervention	Diabetes was associated with lower incidence of fibroid diagnosis	Not assessed

**Table 3:** Data charting of studies on ulipristal acetate for uterine leiomyomas.

Author/Year	Country	Study Design	Sample Size	Intervention and Dosage	Key Findings	Adverse Effects
Donnez et al., 2016 [30]	46 centers in 11 European countries	Double-blind randomized long-term medical management study	451	Duration: 12 weeks, treatment occurred 4 times, UPA 5 mg and 10 mg was used	Fibroid volume decreased in all patients over 73%, heavy menstrual bleeding symptoms show good results in UPA treatment	Headache and hot flushes were among the most commonly reported adverse events.
Ferrero et al., 2016 [31]	Italy	Retrospective study	34 received UPA before surgery	Ulipristal acetate 5 mg/day for 3 months	Reduced intraoperative blood loss, operative time, hemoglobin drop, and postoperative blood transfusion need	Overall complications were not significantly different between groups
Fernandez et al., 2017 [32]	Europe	Prospective study	1473	5 mg/day for 3 months	Reduced fibroid-related clinical burden before planned intervention. Decreased pain and improved quality of life of the patients.	Not specified
Fauser et al., 2017 [33]	4 European countries	Cohort study	64	3 months, 10 mg/day	All endometrial biopsies from the study subjects were benign after 8 cycles of treatment. Median endometrial thickness 10–18 days after the start of menses following treatment courses 5–8 (10–18 days post menses) was 7.0 mm related to the screening time to 9.0 mm	Most frequent adverse events were headache and hot flush.
Luketic et al., 2017 [34]	Canada	Multicenter retrospective study	25 UPA-treated patients	5 mg/day for 3 months	No significant difference in surgical experience between UPA-pretreated patients and untreated patients.	Not clearly reported
Chen et al., 2017 [35]	UK	Retrospective	80	5 mg/day for 3 months	Improvement in their pain score and PMS symptoms	Not specified
Lee et al., 2017 [36]	Korea	Retrospective	51	5 mg/day for 3 months	Fibroid volume reduction was 12.4%,	Not specified
Simon et al., 2018 [37]	USA	Randomized	157 women	12 weeks, 5 mg or 10 mg UPA once a day or placebo group	Improved quality of life and anemia symptoms. UPA receiving 5 mg showed amenorrhea 47.2%, while 10 mg 58.3%, it is good compared with the placebo group.	Hypertension, hot flushes
Liu et al., 2018 [38]	USA, Canada	Randomized, double-blind, placebo-controlled Phase III trial	432	Duration: 12 weeks, UPA 5 mg and 10 mg was used once a day	Compared to placebo, greater improvement was observed in both Uterine Fibroid Symptom and in the Health-Related Quality of Life Revised Activities subscale with 5 mg and 10 mg ulipristal acetate.	Hot flush
Baggio et al., 2018 [39]	Italy	Prospective clinical ultrasound study	91	5 mg/day for 3 months	Uterine volume decreased by 14%, and fibroid volume decreased by 32.8%. Fibroid vascularization also decreased on Doppler ultrasound.	Not specified
Yun et al., 2018 [40]	Korea	Retrospective study	152	5 mg/day for 3 months	Median fibroid volume reduction was 13.1%.	Not reported
Woodhead et al., 2018 [41]	UK	Retrospective study	134	Not specified	80% of patients reported global symptom improvement after the first course. 45.5% reported amenorrhea or light bleeding compared with 4.5% before treatment. Anemia decreased after treatment	Not reported
Brun et al., 2018 [42]	France	Retrospective	66	5 mg/day for 3 months	Clinical success was reported in 54/66 patients (82%), and imaging success, defined as fibroid volume reduction ≥25%, was reported in 39/66 patients (59%).	Not reported
Verguts et al., 2019 [43]	Belgium	Prospective	222	5 mg/day for 3 months	The volume of the fibroids decreased significantly, with the largest measured fibroid decreasing by 50%. Bleeding and pain were significantly decreased with the use of UPA.	Bleeding and blood transfusion
Singh et al., 2018 [44]	Canada	Prospective	75	Not specified	Preoperative UPA did not show a significant improvement or disadvantage during myomectomy across different surgical routes.	Not specified
Lukes et al., 2019 [45]	USA	Randomized trial	589 women	Once a day 5 mg or 10 mg UPA	Symptom severity and health-related quality of life improved in UPA group (5 mg 73.5% and 10 mg 80.6%) compared with placebo group (34.9%)	Hot flushes
Irahara et al., 2020 [46]	Japan	Randomized	121 premenopausal women	Placebo vs. UPA (2,5 mg; 5 mg; 10 mg) once a day. Duration: 12 weeks	Amenorrhea rates were increased in UPA (2.5 mg—60%, 5 mg—72.7%, 10 mg—88%) group compared with placebo (4.5%)	Constipation, headache, nausea, hot flashes, and menorrhagia
Mallick et al., 2019 [47]	UK	Prospective	10	5 mg/day for 3 months	Blood loss was lower in the UPA group	No complications
Osuga et al., 2021 [48]	Japan	Randomized	161 women	UPA 10 mg orally once daily for 12 weeks	In the final treatment week, fibroid volume decreased by up to 23.7%	Headache, menorrhagia, metrorrhagia, genital hemorrhage, and hot flashes
Jha et al., 2021 [49]	India	Prospective clinical study	86	Treatment occurred twice	First treatment revealed fibroid volume decrease of 32%, while after second treatment decreased by 52%	nausea, hot flushes, constipation, and headache

UPA, Ulipristal Acetate.

**Table 4:** Comparative summary of metformin and ulipristal acetate in uterine leiomyoma management.

Category	Metformin	Ulipristal Acetate (UPA)	Ref
Primary Drug Class	Biguanide/AMPK activator and metabolic modifier	Selective Progesterone Receptor Modulator (SPRM)	[25,37]
Main Mechanism of Action	Activates AMPK and causes mTOR inactivation by both TSC2-dependent and TSC2-independent pathways, in addition to local inactivation of aromatase and 17β-HSD.	Competitively binds to progesterone receptors preventing the growth promoting action of progesterone for leiomyoma cells.	[7,24,28,29,37,50,52,54,55]
Effect on Cell Proliferation and Apoptosis	Leiomyoma cell cycle arrested at G0/G1 or S phase and induced apoptosis through increasing the Bax/Bcl-2 ratio. The cells showed decrease in PCNA and Cyclin D1 expression.	Inhibits cell proliferation, thereby it causes an increase in the expression of p21 and p27. It also induces apoptosis through the decrease in the expression of Bcl-2.	[25,51,52,53,54,55]
Effect on Extracellular Matrix (ECM)	Inhibition of TGF-β signaling pathway and decrease in production of matrix metalloproteases (MMP-2/9).	Suppresses the expression of interstitial collagens (Type I/III), fibronectin-1 (FN1), and versican (VCAN); modulates the MMP/TIMP ratio to drive structured ECM degradation.	[53,54,56,57]
Angiogenesis Regulation	Inhibits neo-angiogenesis by decreasing expression of VEGF (Vascular Endothelial Growth Factor) for a lesion.	Local factors that support angiogenesis in leiomyoma are suppressed and the architecture of capillaries is disturbed in leiomyoma tissue	[37,53,54,59]
Clinical Efficacy and Primary Outcomes	Some epidemiological studies showed reduced incidence of leiomyomas, but prospective clinical studies evaluating the drug as a single agent have not been conducted yet.	In terms of clinical efficacy, the drug has promising effects with respect to return to a stable hemodynamic baseline, for the induction of amenorrhea and for allowing of a sustainable reduction in of excess of volume.	[24,28,29,30,31,32,33,34,35,36,37,38,39,40,41,42,43,44,45,46,47,48,49]
Safety Profile and Limitations	The drug has an excellent safety profile supported by long-term follow-up data. Side effects of minor self-limiting gastrointestinal symptoms and lack of data in non-diabetic patients with fibroids.	For the most critical of treatments there is a risk of rare but devastating idiosyncratic drug-induced liver injury (DILI) for which such agents are heavily restricted and require monitoring of liver function.	[18,26,60]
Impact on the Endometrium	It normalizes endometrial tissue, increases insulin sensitivity and, within the endometrium, expresses HOXA10 and GLUT4 without inducing hyperplasia.	They cause benign and fully reversible changes in the endometrium (Progesterone Receptor Modulator-Associated Endometrial Changes, PAEC) instead of hyperplasia.	[54,61,62]

AMPK, AMP-activated protein kinase; mTOR, mammalian target of rapamycin; TSC2, tuberous sclerosis complex 2; 17β-HSD, 17β-hydroxysteroid dehydrogenase; Bax, Bcl-2-associated X protein; Bcl-2, B-cell lymphoma 2; PCNA, proliferating cell nuclear antigen; TGF-β, transforming growth factor beta; MMP-2/9, matrix metalloproteinase-2/9.

**Table 5:** Potential clinical applications of combined therapy in fibroid management.

Clinical Use	Target Population	Clinical Problem	Combined Therapy Role	Expectations	Ref
Preoperative integration	Before surgery in patients with severe bleeding, anemia, or pelvic pressure	Heavy bleeding, anemia, or pelvic pressure	Induces local amenorrhea, stops cell growth, and shrinks fibroid volume	Stabilizes the patient and may allow less invasive surgery	[65,66]
Postoperative integration	After UAE, fibroid ablation, HIFU, or other minimally invasive procedures	Remaining oxygen-deprived fibroid cells may persist	Targets remaining cells, blocks progesterone-driven	May decrease the chance of fibroid regrowth and support better	[1,2,3,4,5,6,7,8]

UAE, uterine artery embolization; HIFU, high-intensity focused ultrasound.

## Data Availability

No new data were created or analyzed in this study. Data sharing does not apply to this article.

## Abbreviations

The following abbreviations are used in this manuscript:

Abbreviation	Definition
AMPK	Adenosine Monophosphate-Activated Protein Kinase
Bcl-2	B-cell Lymphoma 2
Bax	Bcl-2-Associated X Protein
COCs	Combined Oral Contraceptives
DILI	Drug-Induced Liver Injury
ECM	Extracellular Matrix
ELT-3	Eker Leiomyoma Tumor Cell Line
ER-α	Estrogen Receptor Alpha
FN1	Fibronectin-1
GLUT4	Glucose Transporter Type 4
GnRH	Gonadotropin-Releasing Hormone
HIFU	High-Intensity Focused Ultrasound
HOXA10	Homeobox A10
HR	Hazard Ratio
MeSH	Medical Subject Headings
MMP	Matrix Metalloproteinase
NF-κB	Nuclear Factor Kappa B
PAEC	Progesterone Receptor Modulator-Associated Endometrial Changes
PCNA	Proliferating Cell Nuclear Antigen
PCOS	Polycystic Ovary Syndrome
SERMs	Selective Estrogen Receptor Modulators
SPRMs	Selective Progesterone Receptor Modulators
TGF-β	Transforming Growth Factor Beta
TIMP	Tissue Inhibitor of Metalloproteinases
TSC1	Tuberous Sclerosis Complex 1
TSC2	Tuberous Sclerosis Complex 2
UAE	Uterine Artery Embolization
UPA	Ulipristal Acetate
VCAN	Versican
VEGF	Vascular Endothelial Growth Factor
